# Description of the larva of *Oecetis mizrain* Malicky & Graf, 2012 (Trichoptera, Leptoceridae) and *Lepidostoma scotti* (Ulmer, 1930) (Trichoptera, Lepidostomatidae) from Chilimo Forest, Central Ethiopia

**DOI:** 10.3897/zookeys.766.24544

**Published:** 2018-06-13

**Authors:** Yonas Terefe, Simon Vitecek, Wolfram Graf

**Affiliations:** 1 Institute of Hydrobiology and Aquatic Ecology Management, University of Natural Resources and Applied Life Sciences, Vienna, Austria; 2 Department of Biology, College of Natural and Computational Sciences, Ambo University, Ambo, Ethiopia; 3 Department for Limnology & Bio-Oceanography, University of Vienna, Vienna, Austria; 4 Senckenberg Research Institute and Natural History Museum, Frankfurt am Main, Germany

**Keywords:** caddisfly larvae, distribution, ecology, Afrotropical Region, diversity, ecological management

## Abstract

The Ethiopian caddisfly fauna comprises 85 species, including 10 *Oecetis* species and three *Lepidostoma* species. In this context we provide the first species-level descriptions of Ethiopian caddisfly larvae. We describe and illustrate the larvae of *O. mizrain* and *L. scotti*, with additional notes on their habitats and distribution.

## Introduction

Caddisflies are one of the most diverse aquatic insect groups, distributed all over the world. The order Trichoptera comprises approximately 15,000 species (including 685 fossils) in 616 genera and 49 families (Morse 2017). The Oriental region harbors the highest number of species (4865 species), followed by the Neotropical region (2562 species) (de Moor and Ivanov 2008; Holzenthal et al. 2015; Morse 2011). Species numbers in other biogeographic regions such as the Australasian (1439 species), the East Palearctic (1372 species), the Nearctic (1604 species) and the West Palearctic (1888 species) are lower in comparison (Morse 2011). The poorly explored Afrotropical region is currently represented by about 1200 species only, belonging to 28 families and 111 genera (Tobias and Tobias 2008). The Antarctic region is the only biogeographic area where no caddisflies have been recorded (Holzenthal et al. 2015; Morse 2011).

As in many Afrotropical countries, the Ethiopian caddisfly fauna is poorly studied. In a first effort to characterize the almost unknown African caddisfly fauna, Ulmer (1930) described and listed eight Ethiopian Trichoptera species. Three decades later, Kimmins (1963) reported 51 species, of which 17 were described as new. At present, the number has marginally increased to 85 species of 26 genera and nine families (Tobias and Tobias 2008; Morse 2017). Two families, Leptoceridae (35 species in 10 genera) and Hydropsychidae (22 species in six genera), represent over 60% of the currently known Ethiopian Trichoptera diversity.

Caddisflies of the widely distributed genera *Lepidostoma* Rambur, 1842 and *Oecetis* Mclachlan, 1877 comprise 453 and 539 extant species worldwide, respectively (Morse 2017). From Africa and its adjacent islands, 51 *Lepidostoma* and 103 *Oecetis* species are known (Tobias and Tobias 2008). The Checklist of East Africa (Johanson 1992) includes two *Lepidostoma* and 17 *Oecetis* species. Until now, three *Lepidostoma* and 10 *Oecetis* species have been recorded in Ethiopia (Table 1).

**Table 1.** Species of *Lepidostoma* and *Oecetis* occurring in Ethiopia following Kimmins (1963) and Malicky and Graf (2012, 2015); ecoregions according to Abell et al. (2008); distribution status from Tobias and Tobias (2008). AT, Afrotropical; EH, Ethiopian Highlands; LT, Lake Turkana; NER, Northern Eastern Rift; **, not reported outside of Ethiopia.

Taxa	Biogeographic region, Ecoregions (areas)	Distribution in Africa
Genus *Lepidostoma* Rambur, 1842		
*L. missa* Malicky & Graf, 2012	AT, EH (Semien Mts., Chenek pass)	**
*L. scotti* (Ulmer, 1930)	AT, EH (Leliso River, Small stream north of Addis Ababa), LT (Gughe Mt.)	**
*L. zepho* Malicky & Graf, 2012	AT, EH (Leliso River)	**
Genus *Oecetis* Mclachlan, 1877		
*O. armaros* Malicky & Graf, 2015	AT, EH (Small stream N from Addis Ababa)	**
*O. brevis* Kimmins, 1963	AT, LT (Gibe River)	Ghana
*O. brunnescens* (Ulmer, 1923)	AT, NER (Lake Awassa)	Sudan
*O. ghibensis* Kimmins, 1963	AT, LT (Gibe River), NER (Koka Dam, Sodere)	**
*O. mizrain* Malicky & Graf, 2012	AT, EH (Leliso River & Meribo River)	**
*O. montana* Ulmer, 1930	AT, EH (Central Highlands)	**
*O. pangana* Navás, 1930	AT, NER (Koka Dam, Gibe River)	Senegal, Ghana, D.R. Congo
*O. portalensis* Mosely, 1939	AT, EH (Leliso River, Meribo River)	Uganda
*O. setifera* Ulmer, 1922	AT, LT (Lake Awassa, Lake Abaya)	Sudan, D.R. Congo, Malawi, Namibia
*O. tjonnelandi* Kimmins, 1963	AT, LT (Gibe River)	Namibia

Within the genus *Lepidostoma*, the first species described from Ethiopia was *L. scotti* (Ulmer, 1930). Ulmer (1930) described the species based on the material obtained by Hugh Scott and Omur-Cooper during their expedition to the central highlands of Ethiopia. Since then, only two additional species have been described from Ethiopia, *L. missa* and *L. zepho* (Malicky & Graf, 2012). In the genus *Oecetis*, the species described first from Ethiopia was *O. montana* (Ulmer, 1930), based on specimens collected in the central highlands. Later, Kimmins (1963) described *O. tjonnelandi*, *O. ghibensis*, *O. brevis* and indicated the presence of *O. brunnescens* (Ulmer, 1923), *O. montana* (Ulmer, 1930), *O. pangana* Navás, 1930 and *O. setifera* (Ulmer, 1922) in Ethiopia. Most recently, Malicky and Graf (2012, 2015) described *O. armaros* Malicky & Graf, 2015, *O. mizrain* Malicky & Graf, 2012 and reported *O. portalensis* Mosely, 1939 from rivers in the Ethiopian highlands.

Caddisflies are frequently used along with other aquatic fauna as bioindicators in ecological assessment systems as they are sensitive to organic pollution and stream degradation (Barbour et al. 1999). In particular autecological characterization provides vital information for freshwater bioassessment by relating species to ecological conditions (Jones 2008). In this context, species-level identification of bioindicators is of great importance to fully reap the power of ecological analysis (Cranston 1990; Lenat and Resh 2001; Malicky et al. 2008). Species-level determination is achieved for Trichoptera and many other taxonomic groups in Central and Northern European countries where this information is used for stream quality assessments (Hering et al. 2003; Schmidt-Kloiber et al. 2006). Most studies in tropical Africa including Ethiopia, however, are restricted to family-level identiﬁcation due to the lack of taxonomic knowledge (Aschalew and Moog 2015). Therefore, to support efforts to maintain the general ecological health of fresh waters and thus ensure sustainable use of water resources in Ethiopia the compilation of taxonomic and autecological databases for all potential bioindicators is essential. Hence, in this contribution, we describe and illustrate the final instar larvae of *L. scotti* and *O. mizrain* from Ethiopia.

## Material and methods

Larval and adult material was collected at small highland streams (9°4'N, 38°8'E) within Chilimo Forest, in the upper catchment of the Awash River. Association of larvae and adults was enabled by the exclusive occurrence of these *Lepidostoma* and *Oecetis* species at this site and the presence of mature pupae in the case of *O. mizrain*. Chilimo Forest is a dry afromontane forest, located about 97 km west of Addis Ababa and 7 km north of Ginchi town. It covers an area of more than 2500 hectares within an altitudinal range from 2170 m a.s.l. to 3054 m a.s.l. (Teshome and Ensermu 2013). The forest and its surrounding areas receive little precipitation from March to April, while precipitation is highest from June to September with a mean annual rainfall of >1000 mm (Aschalew 2015). The dominant trees species in this forest are *Juniperus procera*, *Podocarpus falcatus*, *Prunus africana*, and *Olea europaea* (Kassa et al. 2009; Teshome and Ensermu 2013).

The collected larval specimens were preserved in 70% ethyl alcohol. Morphological characteristics of specimens were examined and photographed using a Zeiss StereoLumar V.12 equipped with an AxioCamErc5s camera and the Zeiss-native image processing software ZEN. Image series at different focus levels were obtained and stacked via CombineZP (Hadley 2008; Brecko et al. 2014) to create single extended-depth-of-focus images. Larval morphological features and nomenclature of primary setae and setal areas follows Wiggins (1996) and Waringer and Graf (2011).

## Results

### Order Trichoptera

#### Family Leptoceridae

##### Oecetis mizrain

Taxon classificationAnimaliaTrichopteraLeptoceridae

Malicky & Graf, 2012

Figs 1–6 , 7–11 , 12

###### Material.

8 larvae: Ethiopia, Oromia Region, Chilimo forest N of Ginchi, 2451m a.s.l., 9.059719°N, 38.14332°E; 20.ii.2016; leg. & det. W. Graf; specimens deposited in the research collection of W. Graf at the University of Natural Resources and Applied Life Sciences Vienna [contact: wolfram.graf@boku.ac.at] and the Senckenberg Research Institute and Natural History Museum Trichoptera collection [collection number SMFTRI00018576; contact: Steffen U. Pauls – steffen.pauls@senckenberg.de].

###### Description of the 5^th^ instar larva.

*Biometry*. Larva eruciform, body length 4.5–5.0 mm, head width 0.69–0.78 mm (n=4).

*Head*. Head capsule hypognathous, elongated, with smooth surface; head capsule distally slightly narrower. As in all final instar larvae of Leptoceridae, subocular ecdysial line present on parietalia, running from foramen occipitale to lateral section of parietalia, ventrally to eyes, at anterior region of eyes bending dorsally, meeting frontoclypeal suture (Fig. 2, black arrow). General color of head capsule pale to golden brown (Figs 2, 3); posterodorsal margin whitish, ventral occipital margin whitish with pale-brown line; ventral apotome brown with white corners; lateroventral sclerites defined by subocular ecdysial suture dorsally brown; parietalia and frontoclypeus around dorsal ecdysial line with distinct, pale muscle attachment spots; white ring around eyes, slightly wider than eye diameter. Head capsule with complete set of primary setae, and additional linear groups of setae around setae #16 and 17. Frontoclypeus scutiform, without distinct medial constriction. Labrum light brown, with setal brush and all primary setae. Mandible slender, elongate, with single cutting edge and 3 teeth. Ventral apotome isosceles trapezoidal, rounded corners. Antennae near distal parietal border (Fig. 2), long (more than 6× longer than wide) and with single terminal seta (Fig. 2).

**Figures 1–6.**
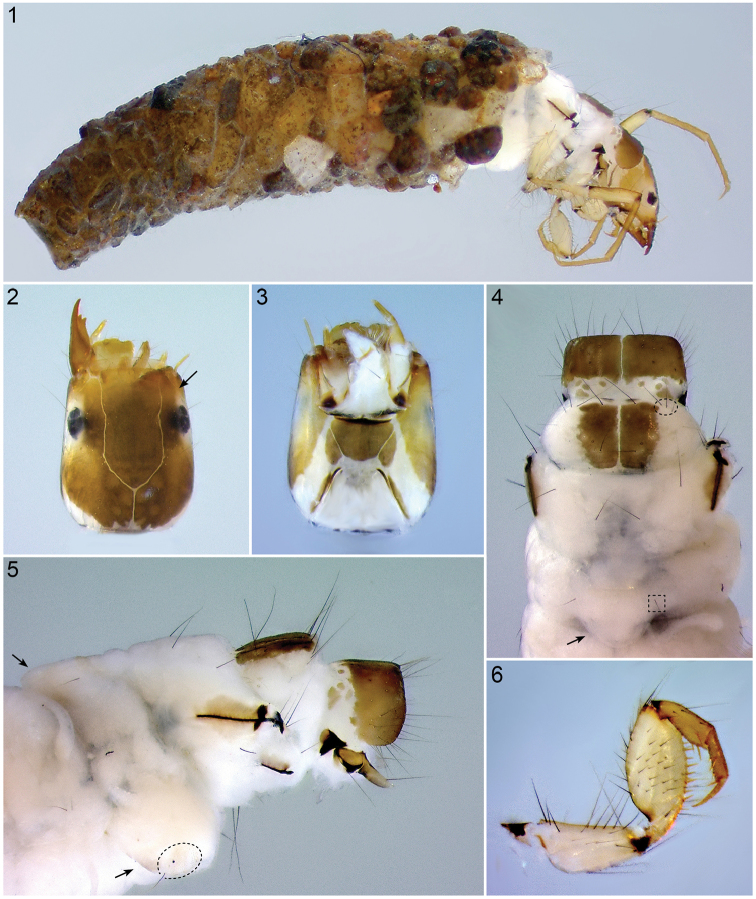
*Oecetis mizrain*, 5^th^ instar larva**. 1** Larva with the case, 14× **2** Head, dorsal view (arrow indicates subocular ecdysial line), 50× **3** Head, ventral view, 65× **4** Thorax (Pro-, Meso- and Metanotum) and abdominal segment I, dorsal view (dashed oval indicates mesonotal *sa_3_*, dashed square indicates adominal segment I *sa_2_*, arrow indicates abdominal segment I dorsal protuberance), 45× **5** Thorax and abdominal segment I, lateral view (arrows indicate abdominal segment I dorsal and lateral protuberances, dashed outline indicates abdominal segment I lateral sclerite), 45× **6** Left front leg, anterior face, 80×.

**Figures 7–11.**
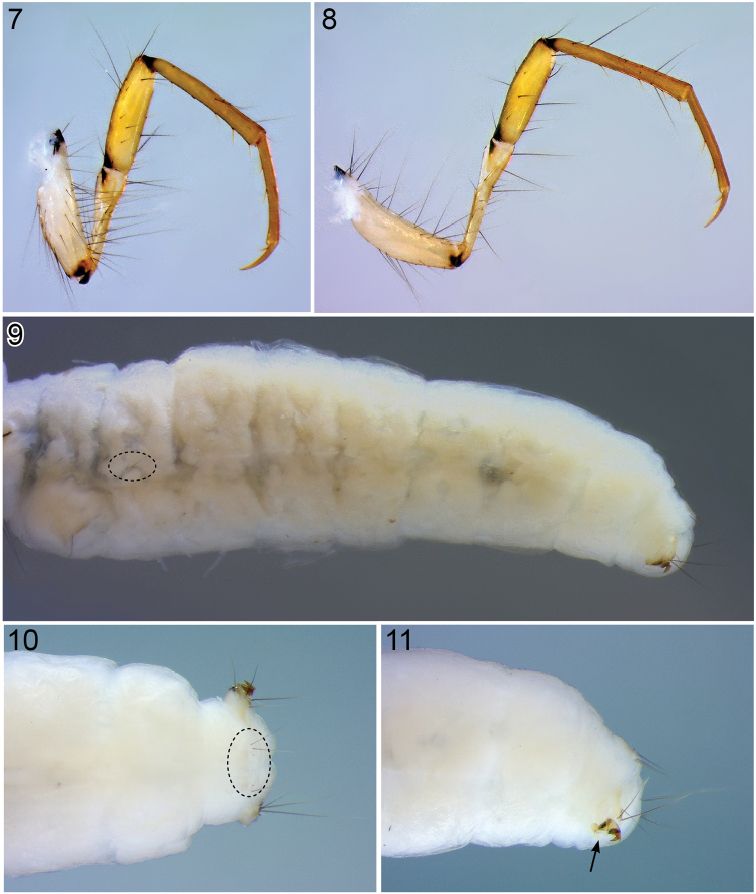
*Oecetis mizrain*, 5^th^ instar larva**. 7** Left middle leg, anterior face, 50× **8** Left hind leg, anterior face, 60× **9** Abdominal segment I–X, lateral view, 25× **10** Abdominal segment IX, dorsal view (dashed oval indicates abdominal segment IX tergite), 50× **11** Anal proleg, lateral view, 50× (arrow indicates section of anal proleg where prominent spines or tines may be present in other Leptoceridae).

*Thorax*. Prothorax fully covered by 2 large sclerites, light brown to brown; small fragments of sclerites present posterior to each pronotal half (Fig. 4). Including anterior setal rows, 25–28 setae of varying lengths distributed over each pronotal half (Figs 4, 5). Mesonotum with 2 large sclerites covering about 50% of its area while not reaching lateral margins, each with small anterior sclerite. Including anterior setal rows, 3–4 setae distributed over each mesonotal half; setae of setal area 3 (*sa_3_*) on the unsclerotized part of each mesonotal half (Fig. 4; dashed oval). Metanotum completely unsclerotized; metanotal setal areas *sa_2_* and *sa_3_* each with single seta only; setal area *sa_1_* not developed (Fig. 4). Metasternum with transverse band of setae on either side, each comprising 2–3 setae (Fig. 5). Legs yellowish beige, with numerous setae (Figs 6–8); foreleg with numerous stout setae on anterior face of femur and with row of stout yellow setae along ventral edge of femur (Fig. 6); foretrochantin with single seta (Fig. 6); tarsal claw of mid leg curved (Fig. 7); hind leg much longer than mid leg, as typical for this genus (Fig. 8).

**Figure 12.**
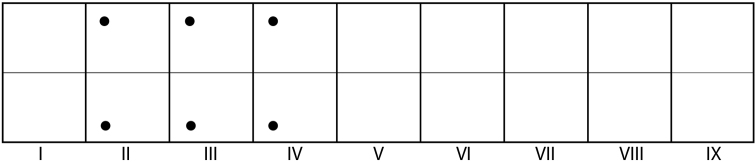
*Oecetis mizrain*, 5^th^ instar larva. Gill diagram indicating position of dorsal and ventral abdominal gills; lateral gills absent. Lateral line seemingly absent.

*Abdomen*. Abdomen white, cylindrical (Fig. 9). Abdominal segment I with 3 protuberances (Figs 4, 5; arrows); dorsal setal areas *sa_1_* and *sa_3_* not developed, dorsal setal area *sa_2_* with single seta on either side (Fig. 4, dashed square); lateral sclerite weakly sclerotized, mostly translucent, oval, with 1 seta (Fig. 5, dashed outline). Abdominal tergum IX with weakly sclerotized, mostly translucent sclerite, bearing 6 long setae in 2 groups concentrated laterally (Fig. 10, dashed oval). Anal proleg weakly sclerotized, each with large lateral sclerite and more strongly sclerotized anal claw and little accessory hook; each bearing several long setae; spines or tines on anal prolegs absent (Fig. 11, arrow). Lateral line not visible, a group of setae (2–3) present laterally on abdominal segment II (Fig. 9, dashed oval); dorsal gills only present at anterolateral position on segments II–IV, lateral gills absent, ventral gills only present at anterolateral position on segments II–IV (Fig. 12).

*Case*. Length 4.9–5.3 mm. Case of final instar larvae constructed of small sand grains, tusk-shaped; anterior opening with overhanging dorsal portion, posterior opening closed with silk (Fig. 1).

#### Family Lepidostomatidae

##### Lepidostoma scotti

Taxon classificationAnimaliaTrichopteraLeptoceridae

Ulmer, 1930

Figs 13–19 , 20–24 , 25

###### Material.

12 larvae: Ethiopia, Oromia Region, Chilimo forest N of Ginchi, 2451m a.s.l., 9.059719°N, 38.14332°E; 20.ii.2016; leg. & det. W. Graf; specimens deposited in the research collection of W. Graf at the University of Natural Resources and Applied Life Sciences Vienna [contact: wolfram.graf@boku.ac.at] and the Senckenberg Research Institute and Natural History Museum Trichoptera collection [collection number SMFTRI00018577; contact: Steffen U. Pauls – steffen.pauls@senckenberg.de].

###### Description of 5^th^ instar larva.

*Biometry*. Larva eruciform, body length 8–9.5 mm, head width 0.87–0.94 mm (n=8).

**Figures 13–19.**
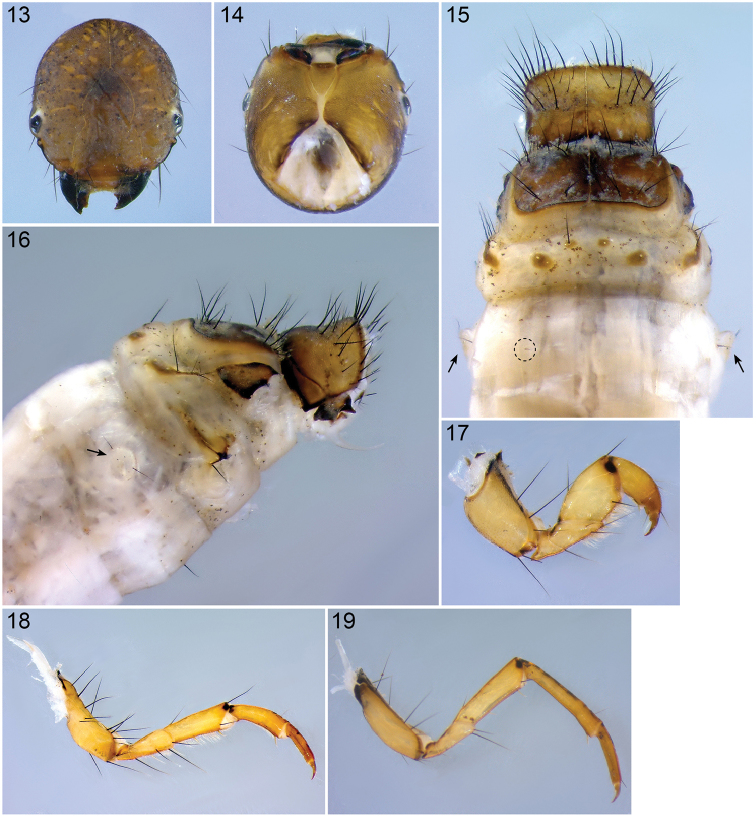
*Lepidostoma scotti*, 5^th^ instar larva**. 13** Head, dorsal view, 40× **14** Head, ventral view, 50× **15** Pro-, Meso- & Metanotum and abdominal segment I, dorsal view (arrows indicate abdominal segment I lateral protuberances, dashed circle indicate abdominal segment I *sa_2_*), 25× **16** Thorax and abdominal segment I, lateral view (arrow indicates abdominal segment I lateral protuberance), 23× **17** Left front leg, anterior face, 50× **18** Left middle leg, anterior face, 40× **19** Left hind leg, anterior face, 40×.

*Head*. Head capsule hypognathous, round; surface granulated, covered in spicules. Color pale to dark brown, with scattered black markings (Fig. 13), area around occipital margin dark brown (Figs 13, 14). Muscle attachment spots pale brown, > 10 on each parietalia, slightly asymmetrical, lining in rows, absent on frontoclypeus. Complete set of 18 primary setae present; setae 4, 5, 13, 15 and 17 very lightly colored; short antenna situated near the anterior margin of each eye, positioned on discrete protuberance (Fig. 13). Whitish ring present around eyes. Frontoclypeus with central constriction; ventral apotome triangular, yellow-brown (Fig. 14). Labrum with 6 pairs of setae, labral brushes at anterior margin present. Mandible robust, black, with 3 teeth.

*Thorax*. Pronotum fully covered by 2 sclerites (Fig. 15), pale brown to brown, at posterior (with small lateral process) and posterolateral margins thickened and darkly lined (Fig. 15); each sclerite bears 20–25 long dark setae mostly concentrated in anterior half (including two rows of setae along anterior margin) (Figs 15, 16). Prosternal horn present, whitish, curved anteriorly (Fig. 16). Mesonotum fully covered by 2 sclerites, brown to dark brown (black near median suture), posterior margin with slightly sclerotized narrow dark line; mesonotal setal areas *sa_1_*, *sa_2_* and *sa_3_* present; *sa_1_* bearing 3–4 setae; *sa_2_* bar-shaped, bearing 6–8 setae, stretching to posterior margin; *sa_3_* bearing 5–6 setae; *sa_1_* and *sa_3_* connected by regular bands of setae (Fig. 15). Metanotum with 6 distinct small sclerotized areas, corresponding to setal areas *sa_1_*, *sa_2_* and *sa_3_*; *sa_1_* and *sa_2_* rounded, each with 1 and 1–2 setae respectively (Fig. 15); *sa_3_* elongate, falcate shaped, bearing 4–6 setae (Figs 15, 16). Thoracic legs yellowish brown, with dark marking at femoro-tibial joint and with dense fringe of setae on ventral edge of coxa, trochanter and femur and long dark setae (Fig. 17); foreleg more robust, shorter than mid- and hind leg; mid leg bearing larger number of setae (majority at the coxa) than fore- and hind legs; tarsal claws similar in all legs, short, robust (Figs 17–19).

**Figures 20–24.**
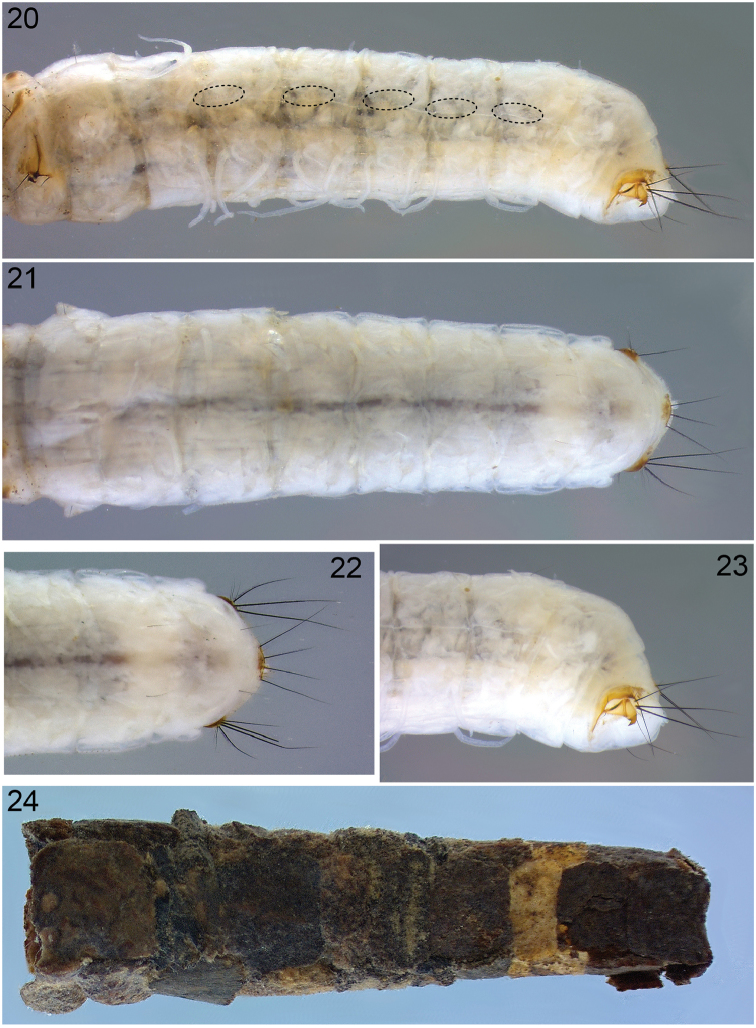
*Lepidostoma scotti*, 5^th^ instar larva**. 20** Abdomen, segments I-X, lateral view (dashed ovals indicate position of forked lamellae), 13× **21** Abdomen, segment I-X, dorsal, 13× **22** Abdominal segment VII-X, dorsal, 25× **23** Abdomen, segment VII-X, lateral, 25×; **24** Larval case, left lateral view, 10×.

**Figure 25.**
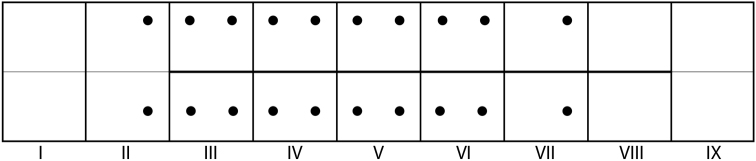
*Lepidostoma scotti*, 5^th^ instar larva. Gill diagram indicating position of dorsal and ventral gills and extent of lateral line (bold line). Lateral gills absent.

*Abdomen*. Color whitish (Figs 20, 21). Abdominal segment I with lateral humps bearing 2 setae (1 on anteroventral margin, 1 on dorsal margin) (Figs 15, 16, 20, 21; highlighted by arrows in Figs 15, 16); dorsal hump absent (Figs 15, 16, 20); dorsal setal areas *sa_1_* and *sa_3_* absent, *sa_2_* present, with a single seta (Fig. 15, dashed circles; Fig. 20); ventral setal areas *sa_2_* and *sa_3_* present, each with a single seta; *sa_1_* absent (Figs 16, 21). Tracheal gills simple, unbranched, as single filaments; dorsal gills on segment II (postsegmental position), segments III–VI (pre- & postsegmental position) and segment VII (postsegmental position); ventral gills on segment II (postsegmental position), segments III–VI (pre- & postsegmental position) and segment VII (postsegmental position); lateral gills absent; position of ventral gills shifted dorsad (Figs 20, 25). Lateral line from segment III–VIII; small bifurcated lamellae dorsal to lateral line present (Figs 20, 25; position of bifurcated lamellae highlighted in dashed ovals). Dorsal sclerite of segment IX semicircular, pale brown, with 4 pairs of setae (outermost lateral and medial setae very long, with interspersed shorter setae) (Fig. 22); anal proleg present, of limnephilid type (Fig. 23).

*Case*. Larval case 9.0–10.5 mm long, constructed from rectangular pieces of plant material; pieces subrectangular to quadratic, parts of barks or leaves; cross-section subrectangular to subquadrangular, tapering towards the posterior end (Fig. 24).

## Discussion

Historically, the most comprehensive faunistic African studies were done in South Africa, Madagascar and West Africa. These studies yielded hundreds of Trichoptera species to the Afrotropical region, of which 253 species are known from South Africa (de Moor and Day 2013), more than 500 species from Madagascar (Benstead et al. 2003), and 343 species were reported from the West Africa region (Tobias and Tobias 2008). However, other regions of the African continent are not as well represented in the available caddisfly literature. In Ethiopia, 85 species are known currently, and the discovery of several new species within the next decades is likely. Particularly, on-going projects on water resource management and capacity building will foster faunistic surveys and thus provide highly relevant baseline data for both taxonomy and ecosystem monitoring.

However, most of the data available on diversity of the African Trichoptera fauna were compiled based on adult specimens, and most species are not known in the larval stage. According to Scott (1986) only 105 of 747 Sub-Saharan species of African Trichoptera were known in the larval stage at that time. Thus, development of more precise biomonitoring tools is severely hampered. Moreover, description of these larval stages could foster development of taxonomic expertise as crucially needed for biomonitoring and sustainable development of freshwater resources in the Afrotropical region. Consequently, faunistic surveys and taxonomic treatment of yet undescribed larval stages are of great value for the development of knowledge and human resources as well as for the documentation of freshwater biodiversity.

In general, larvae of the genus *Lepidostoma* inhabit springs and cool streams that usually have slow water flow and substantial input of allochthonous organic material (Wiggins 2004). Indeed, it is evident from the mandibles of *L. scotti* larvae (Fig. 13) that this species is, like most *Lepidostoma*, a detritivorous shredder that feeds on leaf litter and decaying woody debris. Moreover, larvae of *Lepidostoma* are known to exploit even recalcitrant resources, including the leaves of coniferous plants (Grafius and Anderson 1980; Wiggins 2004). The riparian zone of Chilimo Forest streams is usually dominated by the coniferous plants *Juniperus procera* and *Podocarpus falcatus* (Kassa et al. 2009; Teshome and Ensermu 2013). The leaves of these species most likely represent the primary food source of *L. scotti* larvae in these habitats. Other studies in Ethiopia indicated that *Lepidostoma* larvae exclusively live in forested streams instead of areas with intensified land use (Aschalew 2015; Ferengi 2016).

Larvae of the genus *Oecetis* are found in a wide range of freshwater habitats and are either carnivorous, or behave as detritivores or shredders (e.g., Waringer and Graf 2011). Larvae of carnivorous *Oecetis* species have elongated single-blade mandibles (Waringer and Graf 2011). Based on the elongated bladelike mandibles observed in *O. mizrain*, larvae of this species (Fig. 2) most likely have a predatory feeding behavior.

In addition to information on feeding ecology, stream zonation preference, sensitivity to organic pollution, or sediment load, knowledge on the flight periodicity of potential bioindicators is crucial to determine sampling seasons for biomonitoring approaches. According to Wright et al. (2013), caddisﬂy ﬂight periodicity is likely controlled by a combination of innate behavior and environmental factors, most notably temperature. However, despite repeated sampling efforts in Ethiopia, the flight periods of single species could not yet be defined. Recent collections of adult *L. scotti* and *O. mizrain* revealed that these species were active in October and November (Malicky and Graf 2012, 2015), but their complete flight period remains unclear due to the lack of consistent faunistic surveys. In the eastern Afrotropical Region, these months are considered as the regeneration period for most macroinvertebrates following the heavy rainfall and high flooding season that extends from late June to mid-September (Aschalew 2015). However, flight periodicity of any Ethiopian caddisfly taxa has yet to be studied throughout the year, and further investigations on the annual flight period are currently under way.

Caddisfly larvae are widely used as indicator taxa in freshwater assessments as they exploit a wide range of ecological niches, often are found in abundance and cover a wide sensitivity range (Aschalew and Moog 2015; Barbour et al. 1999; Chakona et al. 2009; Hering et al. 2003). Ideally, assessment of the ecological condition of aquatic ecosystems is based on the identification of macroinvertebrates to species-level (Sharma et al. 2008). Jones (2007) also emphasizes the importance of species datasets for a better interpretation of bioassessment results, as well as testing ecological theories and evaluating threats of extinction to aquatic taxa. In particular, lack of species-level information leads to underestimating the actual differences in community structure (Benstead et al. 2003). Therefore, species-level resolution is fundamental for freshwater biomonitoring (Lenat and Resh 2001; Malicky et al. 2008; Corbi and Trivinho-Strixino 2006).

To achieve a better understanding of Ethiopian freshwater biodiversity and the biogeography of African freshwater fauna in general, description of species and preparation of species-level keys is imperative. Here we provide some data that might be useful for future studies to characterize the Ethiopian caddisfly fauna.

## Supplementary Material

XML Treatment for Oecetis mizrain

XML Treatment for Lepidostoma scotti
